# Management of bone metastases in patients with breast cancer: review of bone-modifying therapy, emerging new agents, and future directions

**DOI:** 10.3389/fonc.2026.1823973

**Published:** 2026-05-08

**Authors:** Gilbert Bader, Brianna Brown, Ashley Pariser Davenport, Margaret Gatti-Mays, Kai CC Johnson, Nerea Lopetegui-Lia, Dionisa Quiroga, Arya Mariam Roy, Sagar Sardesai, Daniel Stover, Robert Wesolowski

**Affiliations:** 1 Division of Medical Oncology, Department of Internal Medicine, The Ohio State University Comprehensive Cancer Center, Columbus, OH, United States; 2 Department of Pharmacy, The Ohio State University Comprehensive Cancer Center, Columbus, OH, United States

**Keywords:** bisphosphonates, bone metastases, breast cancer, clodronate, denosumab, ibandronate, pamidronate, zoledronic acid

## Abstract

Bone metastases occur in 65-75% of patients with metastatic breast cancer. They are often associated with skeletal-related events (SREs), leading to significant morbidity and decline in quality of life (QoL). Treatment with bone-modifying agents is crucial to decrease the risk of SREs and improve QoL. We used PubMed to review key clinical trials that studied the use of bisphosphonates (BPs) and denosumab for the treatment of osseous metastases in patients with metastatic breast cancer. We also reviewed agents that may have the potential to improve SREs. BPs – including clodronate, pamidronate, ibandronate, and zoledronic acid – as well as denosumab have shown efficacy and safety in the treatment of bone metastases secondary to breast cancer. Oral BPs are mainly used in Europe and other countries while zoledronic acid and denosumab are more commonly used in the United States. Zoledronic acid offers the convenience of a once-every-12-week dose. Denosumab may be superior. It is also associated with less renal toxicity and less acute-phase reactions. BPs and denosumab have shown good results; they are usually continued long-term as a palliative treatment. However, they are unlikely to revert osteolytic lesions. There is an unmet need for improvement of SREs. Bone-forming agents should be seriously considered for this purpose.

## 1 Introduction

Bone metastases occur in around 65-75% of patients with metastatic breast cancer (1). They are often associated with skeletal complications referred to as skeletal-related events (SREs). SREs are defined as pathologic fracture, spinal cord compression due to vertebral compression fracture, the need for surgery or radiation to bone (for pain or impending fracture), and hypercalcemia of malignancy (2). Historically, SREs occurred in up to 64% of cases of metastatic breast cancer with osseous metastases prior to the widespread use of bone-modifying therapies such as bisphosphonates (BPs) or denosumab, a RANKL inhibitor (3). SREs are associated with significant morbidity and decline in quality of life (QoL). Osseous breast cancer metastases are predominantly osteolytic, but up to 15-20% can be primarily osteoblastic (4). Patients can have both osteolytic and osteoblastic metastases or mixed metastases harboring both components. The classification of osseous breast metastases into lytic and blastic lesions reflects extremes of a continuum where most lesions contain both elements to some degree. Breast cancer cells activate bone resorption. Factors secreted by breast cancer cells, primarily parathyroid hormone-related peptide (PTHrP), increase the expression of RANKL on stromal cells and osteoblasts. Binding of RANKL to its receptor RANK expressed on the surface of osteoclast precursors induces the formation of osteoclasts (5).

**Figure 1:**
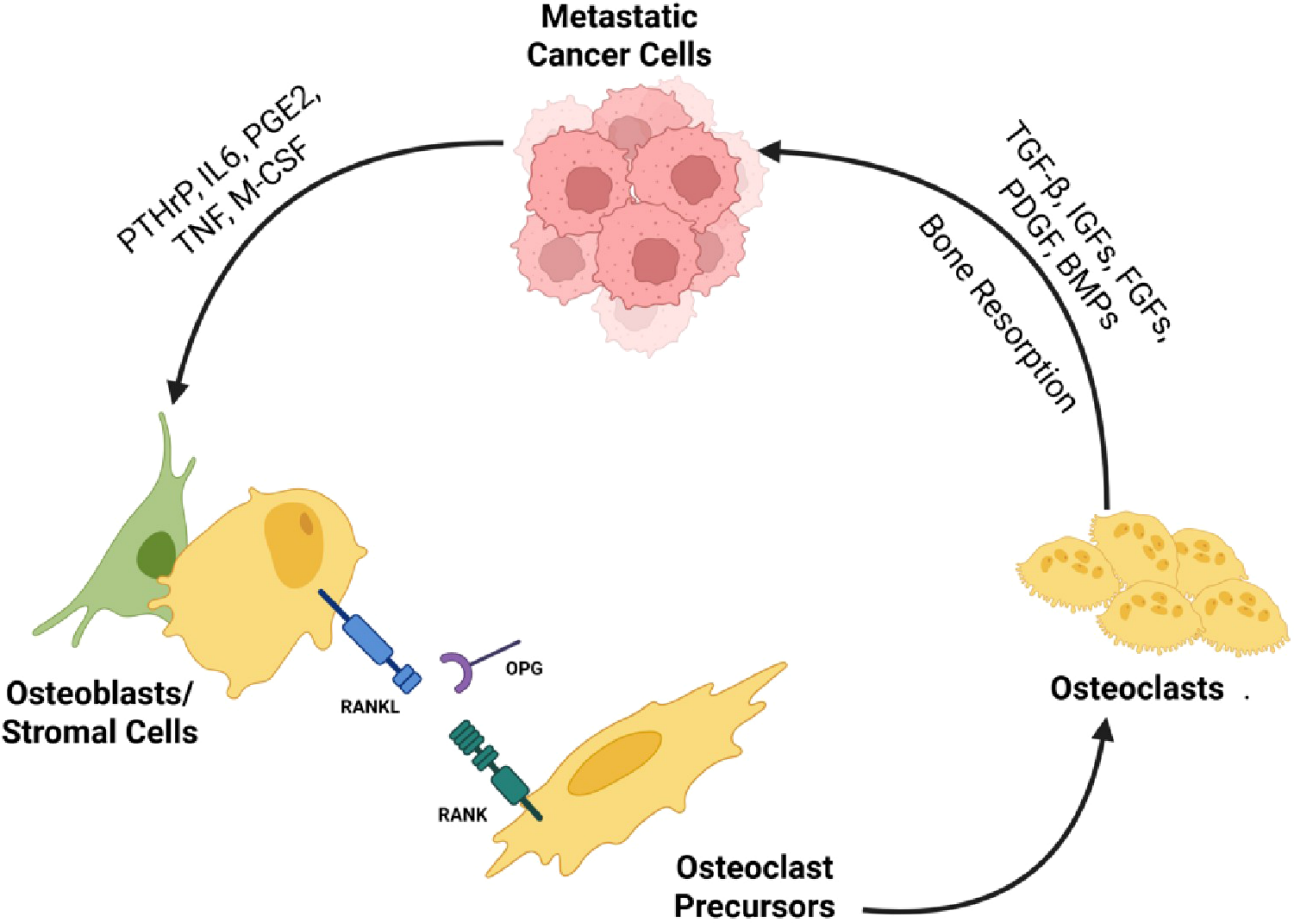
Mechanism of bone metastasis: key factors.

Osteoprotegerin (OPG) is a decoy receptor of RANKL. It is expressed by osteoblasts and stromal cells, impairs the interaction of RANKL with RANK, and therefore inhibits the differentiation of osteoclasts. The ratio of RANKL to OPG regulates osteolysis (6). Bone resorption releases factors such as insulin-like growth factors (IGFs), fibroblast growth factors (FGFs), and transforming growth factor beta (TGF-β), which in-turn enhance tumor growth and increase the production of PTHrP; this leads to a vicious cycle of tumor growth and bone resorption (5, 7) (See **Figure 1**).

In addition to increasing bone resorption, breast cancer cells inhibit osteoblast differentiation. Canonical Wnt signaling is a major pathway for promoting osteoblast differentiation. Sclerostin is a soluble Wnt antagonist that prevents binding of Wnt ligands to low-density lipoprotein receptor-related proteins 5 and 6 (Lrp5/6), inhibiting the Wnt pathway (8). Dickkopf1 (DKK1) is also a Wnt-signaling antagonist. Breast cancer cells have been shown to express sclerostin and DKK1, suppressing osteoblast differentiation (9, 10). The mechanism of osteoblastic metastasis is not well understood. Endothelin-1 may play a role in osteoblastic breast cancer metastases (11). It is suggested that bone resorption precedes the formation of osteoblastic metastases (12). A cycle where osteoblasts release growth factors that stimulate cancer cells may be present, similar to the cycle seen in osteolytic lesions (5). Although bone mass is increased overall, the structure and integrity of the focal bone lesion is impaired, resulting in increased bone fragility (13). In osteoblastic metastasis, osteoblasts form irregular trabecular woven bone, prone to low-energy fracture.

## 2 Treatment of metastatic skeletal lesions in patients with metastatic breast cancer

Bone anti-resorptive drugs (also known as bone-modifying therapy) have shown safety and efficacy in treating bone metastases, with the main benefit being a reduction in SREs. BPs and denosumab (antibody-blocking RANKL) have been approved and are commonly used in this setting. In this section, we review bone-modifying agents approved for use in patients with breast cancer who have skeletal metastases.

### 2.1 Approved agents

#### 2.1.1 Bisphosphonates

##### 2.1.1.1 Clodronate

One of the first BPs that demonstrated benefit in patients with breast cancer with bone metastases was oral clodronate.

A randomized controlled trial enrolled 173 patients with bone metastases due to breast cancer to receive 1600 mg/day of oral clodronate or placebo for 18 months (14). The study was later extended to 3 years. It was the first large, double-blind, placebo-controlled trial that examined the effect of an oral BP on SREs secondary to osseous metastases in breast cancer. Clodronate was associated with a 27% reduction in the overall bone events rate (*P* < 0.001). A trend to longer time to first SRE was also seen; however, it was not statistically significant. No overall survival (OS) benefit was observed. The greatest effect in reduction of fracture was seen in the axial skeleton rather than the appendicular skeleton.

An additional controlled study randomized 144 patients with bone metastases secondary to breast cancer to receive either 1600 mg/day of clodronate or placebo for up to 12 months (15). The time to first SRE was 244 days with clodronate and 180 days with placebo (*P* = 0.05).

This agent is not approved for human use in the United States (US). However, it has been in use in Europe and other countries. In fact, the main reason it is not approved in the US is regulatory and commercial. A new drug application was not submitted to the FDA by the manufacturer likely due to the availability of new BPs at that time in the US such as pamidronate and zoledronic acid along with safety concerns such as elevation in transaminases.

##### 2.1.1.2 Pamidronate

The Protocol 19 Aredia Breast Cancer Study Group enrolled 382 patients with metastatic breast cancer with at least 1 lytic osseous lesion to receive 90 mg of pamidronate intravenously (IV) once a month versus placebo for 12 months (16). All patients were receiving chemotherapy at enrollment. The median time to first SRE was 13.1 months in patients treated with pamidronate versus 7 months in patients treated with placebo (*P* = 0.005). The percentage of SREs was 43% and 56% in the pamidronate and placebo groups, respectively (*P* = 0.008). The only SRE for which incidence was not reduced with pamidronate was pathologic vertebral fracture. This study was extended for a second year (17). With longer time on treatment, the median time to first SRE was 13.9 months in the pamidronate group versus 7 months in the placebo group (*P* < 0.001). The percentage of patients who experienced SREs was 42-57% and 63-76% with pamidronate and placebo, respectively (*P* < 0.001). More patients in the pamidronate arm had complete or partial response in terms of bone metastases. No statistically significant OS benefit was observed.

The Protocol 18 Aredia Breast Cancer Study Group enrolled 372 patients to receive 90 mg of pamidronate via IV every 4 weeks for 24 months or placebo (18). In contrast with Protocol 19, the patients were receiving endocrine therapy at enrollment. The percentage of SREs was 56% and 67% in the pamidronate and placebo arms, respectively (*P* = 0.027). The median time to first SRE was 10.4 months with pamidronate compared to 6.9 months with placebo (*P* = 0.049). No difference in OS or bone metastases response rate was observed between arms.

Outcomes observed in the Protocol 19 study seem to be better than the outcomes seen in the Protocol 18 study. In fact, patients enrolled in Protocol 19 likely had more aggressive disease since they were receiving chemotherapy at enrollment. Thus, the suppression of bone resorption likely had a greater effect on tumor growth, leading to better outcomes compared to Protocol 18. Another contributing factor is likely the effect of aromatase inhibitors on bone density. Studies have shown increased fracture risk with the use of adjuvant aromatase inhibitors (19–21). The rate of fracture was 2.93% with anastrozole over 100 months, 8.6% with letrozole over 60 months, and 4.3% with exemestane over 55 months.

Follow-up data from combined Protocols 19 and 18 were subsequently reported (3). Skeletal morbidity rate (SMR) was adopted as a primary endpoint. SMR (events/year) is the ratio of the number of SREs divided by the time on trial. The SMR at the end of 24 cycles was 2.4 skeletal complications/year with pamidronate versus 3.7 with placebo (*P* < 0.001). The percentage of SREs was 51% with pamidronate and 64% with placebo (*P* < 0.001). The median time to first SRE was 12.7 months with pamidronate and 7 months with placebo (*P* < 0.001). Thirty-two percent of patients in the pamidronate group and 22% of patients in the placebo group had complete or partial response in osseous metastases (*P* = 0.002). Pain was worse in the placebo group. There was no statistically significant difference in OS.

##### 2.1.1.3 Ibandronate

A phase 3 study enrolled 466 patients to receive 2 mg or 6 mg of IV ibandronate given every 3–4 weeks for up to 2 years or placebo (22). Skeletal morbidity period rate (SMPR) was used as the primary efficacy endpoint. SMPR is the number of 12-week periods with new skeletal complications. SMPR is the number of periods with new skeletal events +1 divided by the number of 12 week-periods +0.5. It may be a better endpoint to use than SMR since skeletal complications close together can sometimes be related to a singular event, such as pathologic fracture leading to surgery and radiation, rather than separate events. Therefore, it may be more accurate to count skeletal complications occurring within a single 12-week period as 1 occurrence. SMPR was 20% lower with 6 mg of ibandronate (*P* = 0.004) compared to placebo. No significant benefit was observed with 2 mg of ibandronate. There was a significant decrease in vertebral fractures and a nonsignificant decrease in nonvertebral fractures. The median time to first SRE was 50.6 weeks with 6 mg of ibandronate and 33.1 weeks with placebo (*P* = 0.018).

Subsequently, the results of 2 pooled phase 3 studies using oral ibandronate were reported (23). They enrolled 564 patients to receive 50 mg of oral ibandronate versus placebo for 96 weeks. There was a 19% reduction in mean SMPR associated with oral ibandronate (*P* = 0.004). The median time to first SRE was 90.3 weeks in the oral ibandronate group and 64.9 weeks in the placebo group (*P* = 0.089). There was no significant difference in the number of fractures. When the 96-week period ended, patients were allowed to either continue or start ibandronate for another 96 weeks (24). The role of this extension study was to determine the tolerability of oral ibandronate for up to 4 years. There was no evidence of cumulative toxicity, and no adverse renal events were observed.

A similar extension phase was performed for IV ibandronate (25). After the initial 96-week period of a phase 3 study, 62 patients received 6 mg of IV ibandronate in order to assess its long-term safety and tolerability. Fewer treatment-related adverse events were reported in the extension phase than in the initial phase, and serum creatinine (Cr) remained stable.

##### 2.1.1.4 Zoledronic acid

An international, randomized, double-blind study compared 4 mg and 8 mg of IV zoledronic acid to 90 mg of IV pamidronate. Treatment was given every 3–4 weeks for 12 months (26). Subsequent amendment of the study required patients who were receiving 8 mg of zoledronic acid to switch to the 4 mg dose due to concerns over renal safety. The study enrolled 1130 patients. The proportion of patients with SREs was similar between treatment groups. Among patients with no lytic lesions, zoledronic acid was comparable to pamidronate. Within the subgroup of patients with at least 1 lytic lesion, the percentage of patients with SREs was 48% in the zoledronic acid group and 58% in pamidronate group (*P* = 0.058). In addition, within the lytic subgroup, the median time to first SRE was 310 days with zoledronic acid compared to 174 days with pamidronate (*P* = 0.013). There was also a 30% risk reduction of skeletal events in the lytic subgroup (20% reduction in the whole zoledronic acid group). The study showed that zoledronic acid was more effective than pamidronate in patients with at least 1 lytic lesion. Thus, at that time zoledronic acid was considered the agent of choice for osseous metastases secondary to breast cancer.

Since BPs bind to hydroxyapatite and remain in the bone for years, the ZOOM trial explored the option of giving zoledronic acid every 12 weeks instead of every 4 weeks (27). It was a phase 3, randomized, open-label, noninferiority trial that enrolled patients who were treated with zoledronic acid every 3–4 weeks for 12–15 months before entering the study. Zoledronic acid (4 mg) was given every 12 weeks (experimental arm) or every 4 weeks (control arm) for 1 year. The study enrolled 425 patients. SMR was 0.26 in the 12-week group and 0.22 in the 4-week group. The 12-week schedule was noninferior to the 4-week schedule. Multiple event analysis didn’t show significant difference between groups. The median time to first SRE could not be calculated due to a very low event rate. The percentage of adverse events was < 1% in the 12-week group and 1% in the 4-week group. Jaw necrosis occurred in 2% of patients in the 12-week group compared to 1% in the 4-week group. N-terminal telopeptide (NTX) level, a marker of bone resorption, increased in the 12-week group and remained stable in the 4-week group, suggesting a partial recovery of bone resorption in the 12-week group. That increase in NTX level raised concern about long-term reduction in SMR due to recovery of bone resorption.

OPTIMIZE-2 was a prospective, randomized, double-blind trial that enrolled patients to receive 4 mg of zoledronic acid given every 4 weeks versus every 12 weeks for 1 year (28). The patients previously received 9 or more doses of pamidronate or zoledronic acid before enrollment. The study enrolled 416 patients. Twenty-two percent of patients in the 4-week group and 23.2% of patients in the 12-week group developed at least 1 SRE (*P* = 0.02). The 12-week dose was noninferior to the 4-week dose. The time to first SRE and SMR were not statistically different between groups. Safety profiles and bone markers were also similar between groups.

In ZOOM and OPTIMIZE-2, the patients were pretreated with 4 mg of zoledronic acid every 4 weeks for 9–15 months before randomization. The CALGB 70604 trial randomized patients upfront to receive zoledronic acid every 4 weeks or 12 weeks for 2 years (29). The study enrolled 855 patients. The patients had at least 1 bone metastasis from breast cancer, prostate cancer, or multiple myeloma at enrollment. The percentage of patients who developed at least 1 SRE in 2 years was 29.5% in the 4-week group and 28.6% in the 12-week group, indicating noninferiority of the 12-week group (*P* < 0.001). Pain scores were similar in both arms. Jaw osteonecrosis and grade 3 or 4 elevation in creatinine were not statistically different between both groups. C-terminal telopeptide (CTX) levels were significantly lower in the 4-week group. However, the difference in the degree of suppression of CTX was not clinically significant because the incidence of SREs was similar. Compliance was better in the 12-week group: 63% of patients didn’t have treatment delay compared to 38% in the 4-week group. The median time to first SRE was 15.7 months in the 4-week group and 16.8 months in the12-week group.

A systematic review of randomized clinical trials comparing BPs to placebo or other BPs reinforced the role of BPs as a main treatment of bone metastases from breast cancer (30). In that review, 9 studies including 2189 women with osseous metastases from breast cancer showed a 17% decrease in the risk of SREs associated with BPs (risk ratio [RR] 0.83, confidence interval [CI] 0.78-0.89, *P* < 0.00001). The relative risk was 0.59 in patients treated with zoledronic acid, 0.77 with pamidronate, and 0.84 with clodronate. BPs didn’t affect OS, nor did they reduce the incidence of SREs in women without osseous metastases. **Tables 1** and **2** provide a summary of bisphosphonate studies.

**Table 1:** Summary of bisphosphonate studies.

Agent	Study	Year	Patients	Outcomes
Clodronate	Paterson et al. (14) Randomized, double-blind, placebo controlled trial	1993	173 patients with bone metastases due to breast cancer	• 27% reduction in bone events • Trend to longer time to first SRE • No OS benefit • Main benefit in axial skeleton
	Tubiana-Hulin et al. (15) Randomized, double-blind, placebo controlled trial	2001	144 patients with osteolytic bone metastases due to breast cancer	• Time to first SRE 244 days with clodronate vs. 180 days with placebo
Pamidronate	Hortobagyi et al. (16) Randomized, double-blind, placebo controlled trial	1996	382 patients with at least 1 lytic lesion due to breast cancer and receiving chemotherapy	• Median time to first SRE 13.1 vs. 7 months • SRE 43% vs. 56.1% • No reduction in vertebral fracture
	Hortobagyi et al. (17)	1998	Previous study was continued for 2 years	• Median time to first SRE 13.9 vs. 7 months • SRE 42-57% vs. 63-76% • No OS benefit
	Theriault et al. (18) Randomized, double-blind, placebo controlled trial	1999	372 patients with at least 1 lytic lesion due to breast cancer receiving endocrine therapy	• Median time to first SRE 10.4 vs. 6.9 months • SRE 56% vs. 67% • No OS or bone response benefit
	Lipton et al. (3) Follow-up results from combined protocols 19 and 18	2000	751	• Median time to first SRE 12.7 vs. 7 months • SRE 51% vs. 64% • SMR 2.4 vs. 3.7
Ibandronate	Body et al. (22) Randomized, double-blind, placebo controlled, phase 3 trial	2003	466 patients with bone metastases due to breast cancer	• SMPR 20% lower with 6-mg, decrease in nonvertebral fracture is non-significant • Median time to first SRE 50.6 vs. 33.1 weeks
	Body et al. (23) Randomized, double-blind, placebo controlled, phase 3 trial	2004	564 patients with bone metastases due to breast cancer	• 19% reduction in SMPR • No difference in number of fractures • Median time to first SRE 90.3 vs. 64.9 weeks
	McLachlan et al. (24)	2006	Extension study	• No cumulative toxicity
	Pecherstorfer et al. (25)	2006	Extension study	• Fewer AEs, no renal AE, Cr stable
Zoledronic acid	Rosen et al. (26) Randomized, double-blind trial	2003	1130 patients with at least 1 bone metastasis due to breast cancer	• In lytic group: SRE 48% vs. 58%, median time to first SRE 310 vs. 174 days • Multiple-event analysis: 30% reduction in skeletal events

**Table 2:** Zoledronic acid every 4 weeks vs every 12 weeks.

Agent	Study	Year	Patients	Outcomes
Zoledronic acid	Amadori et al. (27) Phase 3, open-label, randomized, non-inferiority trial	2013	425 patients with bone metastases due to breast cancer	• SMR 0.22 in 12-week group vs. 0.22 in 4-week group, noninferior • Similar AEs, partial recovery in bone resorption
	Hortobagyi et al. (28) Phase 3, double-blind, randomized trial	2017	416 patients with bone metastases due to breast cancer, previously received standard dosing regimen of zoledronic acid or pamidronate	• SRE 23.2% in 12-week group vs. 22% in 4-week group, noninferior • Time to first SRE and SMR not different • Safety profile and markers similar
	Hinelstein et al. (29) Randomized, open-label, non-inferiority trial	2017	1822 patients with bone metastases due to breast cancer, prostate cancer or multiple myeloma. 855 patients with breast cancer	• SRE 28.6% in 12-week group vs. 29.5% in 4-week group, noninferior • Median time to first SRE similar • Pain scores, osteonecrosis, increase in Cr similar • Better compliance • Less suppression of bone resorption

#### 2.1.2 Denosumab

Denosumab is a monoclonal antibody directed against RANKL; it is the only US Food and Drug Administration (FDA)-approved inhibitor of RANK signaling. Unlike BPs, it is given subcutaneously and does not accumulate in the bone. A randomized, double-blind, controlled study was performed to determine the safety and efficacy of denosumab in 25 patients with multiple myeloma and 29 patients with breast cancer with osseous metastases (31). The patients were assigned to receive a single dose of denosumab or 90 mg of pamidronate. The denosumab dose was sequentially escalated from 0.1 mg/Kg to 0.3, 1, and 3 mg/Kg. In the breast cancer stratum, the urinary NTX (uNTX) level was significantly reduced as early as 1 day after a single dose of denosumab or pamidronate. The duration of suppression of uNTX was dose dependent in the denosumab cohort. The suppression of uNTX was more sustained with denosumab than with pamidronate. It persisted up to 84 days after a dose of 1 or 3 mg/Kg of denosumab. Bone resorption resumed 3–4 weeks after pamidronate. Denosumab was well tolerated.

A phase 2 randomized, controlled study investigated the efficacy and safety of 5 dosing regimens of denosumab in patients with osseous metastases from breast cancer and compared the results with those of patients treated with IV BPs (32). The patients were assigned to receive 30, 120, or 180 mg of denosumab every 4 weeks or 60 or 180 mg given every 12 weeks or IV BPs including zoledronic acid, pamidronate, or ibandronate given every 4 weeks. The study enrolled 255 patients. The suppression of uNTX/Cr reached the nadir after the first dose of denosumab. The suppression was sustained in patients who received denosumab every 4 weeks but not in all patients who received denosumab every 12 weeks. More patients in the denosumab cohorts achieved >65% reduction in uNTX/Cr compared to patients in the BP cohort. Hypocalcemia was seen in 8% of patients who received denosumab and 5% in patients who received BPs. Most common adverse events seen in patients who received IV BP were pyrexia, arthralgia, asthenia, bone pain, headache and vomiting. Most common side effects seen in patients who received denosumab were asthenia, nausea and vomiting. Osteonecrosis of the jaw was not reported in this study. No serious adverse events were attributed to denosumab or IV BP. A pharmacokinetics (PK)/pharmacodynamics (PD) simulation suggested that the dose of 120 mg every 4 weeks would result in suppression of uNTX/Cr in 95% of patients.

A randomized, phase 2, open-label study enrolled patients with bone metastases from prostate cancer, breast cancer, or other neoplasms who had elevated uNTX levels despite IV BP therapy (33). The patients were randomized to continue BP every 4 weeks or receive 180 mg of denosumab every 4 weeks or every 12 weeks. The median percent reduction in uNTX at week 13 was 78% in patients treated with denosumab and 33% in those treated with BP. The percentage of patients who maintained uNTX suppression was 64% in the denosumab group and 37% in BP group (*P* = 0.01). The uNTX nadir occurred faster in patients treated with denosumab compared to the control arm. Escape from suppression was observed in more patients who continued BP or received denosumab every 12 weeks. Denosumab was shown to be able to further suppress bone resorption in patients who were already taking BPs. The rates of adverse events were similar. The most common adverse events seen in patients who received IV BP were bone pain, asthenia, nausea and vomiting. The most common adverse events seen in patients who received denosumab were bone pain, asthenia, nausea and constipation. Osteonecrosis of the jaw was not reported.

A phase 3, randomized, double-blind, controlled study enrolled patients to receive 120 mg of denosumab and IV placebo every 4 weeks versus 4 mg of zoledronic acid with subcutaneous placebo every 4 weeks (34). It was a noninferiority trial with a secondary objective of superiority. Thus, the superiority results should be interpreted with caution. The study enrolled 1960 patients. Denosumab was superior: it delayed time to first SRE by 18% (*P* < 0.001 noninferiority, *P* = 0.01 superiority). The median time to first SRE was 26.4 months for zoledronic acid and was not reached for denosumab. The latter reduced the risk of developing multiple SREs by 23% compared to zoledronic acid (*P* = 0.01) and decreased mean SMR by 22% (*P* = 0.004). No difference between OS and disease progression was seen.

Denosumab caused more pronounced suppression of bone resorption. At week 13, uNTX/Cr decreased by 80% with denosumab compared to 68% with zoledronic acid (*P* < 0.001). Severe and serious adverse events were similar in both groups. Acute-phase reactions within the first 3 days after treatment were 2.7 times more common with zoledronic acid. Renal toxicity occurred in 4.9% of patients who received denosumab and 8.5% of patients who received zoledronic acid. The percentage of patients who developed jaw osteonecrosis was 2% with denosumab and 1.4% with zoledronic acid; the difference was not significant. **Table 3** provides a summary of denosumab studies.

**Table 3:** Summary of denosumab studies.

Agent	Study	Year	Patients	Outcomes
Denosumab	Body et al. (31) Randomized, double-blind, controlled trial	2006	Patients with bone metastases due to breast cancer or multiple myeloma. 29 patients had breast cancer	• NTX suppression more sustained with denosumab compared to pamidronate
	Lipton et al. (32) Phase 2, randomized, controlled trial.	2007	255 patients with bone metastases due to breast cancer	• Denosumab 120 mg every 4 weeks more effective in reducing uNTX/Cr compared to BP
	Fizazi et al. (33) Phase 2, randomized, open-label trial	2009	111 patients with bone metastases due to breast cancer, multiple myeloma or other cancers with uNTX > 50 nmol/L BCE/nM Creat despite IV BP	• Denosumab can further suppress bone resorption in patients already taking BP.
	Stopeck et al. (34) Randomized, double-blind, controlled trial	2010	1960 patients with bone metastases due to breast cancer	• Denosumab delayed time to first SRE and reduced risk of SREs compared to zoledronic acid.

## 3 Potential novel options

### 3.1 Anti-sclerostin monoclonal antibody

Setrusumab, an anti-sclerostin antibody, has shown good outcomes in a preclinical study in an animal model of bone metastases from breast cancer (9). This study showed that tumor growth in bones was significantly reduced in mice that received setrusumab. Tumor reduction was confirmed by histological analysis of metastases. In addition, anti-sclerostin antibody therapy didn’t increase extraskeletal metastases and was associated with a survival benefit. It also increased the trabecular bone mass of the distal femur and the proximal tibia as well as the cortical thickness of the femoral midshaft. It showed the capacity to restore bone formation inhibited by breast cancer. Amino propeptide of type 1 collagen (P1NP), a biomarker of bone formation, was higher, and tartrate-resistant acid phosphatase 5b (TRAP5b), a biomarker of bone resorption, was lower in the anti-sclerostin antibody arm compared to the control arm. The number and size of osteoclasts were reduced, and the number and size of osteoblasts were increased in the experimental group compared to the control group. Thus, data indicates that anti-sclerostin antibody treatment increases bone mass by restoring the osteoblast function and decreasing tumor-mediated osteolysis, thereby reverting osteolytic lesions.

Romosozumab, an anti-sclerostin monoclonal antibody, is already FDA approved for osteoporosis treatment. In a phase 2 randomized placebo-controlled trial, romosozumab given subcutaneously was studied in postmenopausal women with osteoporosis. It was associated with increased bone density in the lumbar spine, total hip, and femoral neck (35). The increase in bone density was greater with romosozumab compared to alendronate or teriparatide. The largest gains were observed with a 210-mg monthly dose, given subcutaneously every month for 12 months. The increase in bone formation markers was transient. However, the decrease in bone resorption markers was longer lasting. Romosozumab caused a rapid and marked, but transient, increase in bone formation and a moderate but more sustained decrease in bone resorption. In a phase 3 trial, postmenopausal women with osteoporosis were randomized to receive romosozumab or placebo for 1 year followed by denosumab for 1 year (36). The risk of new vertebral fracture was 73% lower in the romosozumab arm compared to placebo, and the risk of clinical fracture (non-vertebral and symptomatic vertebral) was 36% lower at 1 year. The cumulative 2-year incidence of new vertebral fracture was lower in the arm of patients who originally received romosozumab (0.6% vs 2.5%). Bone density continued to increase in the romosozumab arm after transition to denosumab.

Romosozumab also showed efficacy in treating male patients. It increased bone density in the lumbar spine and hips (37). In 2019, the FDA approved romosozumab for osteoporosis treatment in postmenopausal women at high risk for fracture. The most common side effects of this medication include arthralgia, headache, and hypersensitivity reaction. Romosozumab was shown to be non-carcinogenic in rats (38). It is being studied in patients who have multiple myeloma and osteoporosis (39). In addition, in a prospective exploratory study in postmenopausal women with smoldering myeloma, romosozumab was shown to improve bone density without evidence of disease progression over a 12-month period (40). Whether romosozumab has the potential to reduce SREs in patients with bone metastases from breast cancer remains to be investigated.

### 3.2 Nerve growth factor monoclonal antibody

Tanezumab, a monoclonal antibody against nerve growth factor, was studied in combination with opioids to treat osseous metastases due to prostate cancer, breast cancer, multiple myeloma, or renal cell carcinoma (41). Study 1003 was a randomized, double-blind, placebo-controlled, phase 2 study. It randomized 59 patients with bone metastases due to prostate cancer, breast cancer, renal cell carcinoma, or multiple myeloma, who were on daily opioids for bone metastasis-related pain. Study 1029 was an extension. No statistically significant difference in the change from baseline in daily average pain was observed. *Post Hoc* analysis showed that tanezumab had greater efficacy in patients with higher baseline pain or lower baseline opioid use. Larger studies are needed to further explore the role of tanezumab in alleviating osseous metastasis-related pain.

### 3.3 Soluble LGR4 extracellular domain

Leucin-rich repeat-containing G protein-coupled receptor 4 (LGR4) is a receptor for RANKL. The soluble LGR4 extracellular domain (ECD) binds RANKL and inhibits osteoclast differentiation *in vivo* (42). It is a potential novel anti-resorptive agent. It remains in preclinical development.

## 4 Discussion

Bone anti-resorptive treatments including BPs and denosumab have shown good results in treating osseous metastases secondary to breast cancer and are currently widely used as standard-of-care agents. Clinical trials studied the use of bone-modifying agents for a limited duration. However, it is believed that longer treatment duration may provide additional benefit. Bone-modifying agents are typically continued long-term as a palliative treatment for patients with bone metastases. Parenteral agents are more commonly used in the US. Denosumab is widely used and may be more effective than BPs. Zoledronic acid may be a good alternative for patients who desire less frequent doses or have financial limitations.

Although bone anti-resorptive drugs have established an important role in the treatment of osseous metastases, they can’t revert osteolytic lesions. Thus, there is a clear unmet need for treatments that can stimulate osteoblast function with the intent to restore bone integrity without increasing the risk of malignancies. Sclerostin inhibition seems to have great potential to satisfy this need. Many agents are in the early stages of development. Bone-forming agents should be seriously considered for this purpose. Studies to investigate the risk/benefit ratio of those agents in breast cancer patients with bone metastases are needed.
